# The Frequency and Spectrum of Chromosomal Translocations in a Cohort of Sri Lankans

**DOI:** 10.1155/2019/9797104

**Published:** 2019-04-02

**Authors:** Chamara S. Paththinige, Nirmala D. Sirisena, U. G. I. U. Kariyawasam, Vajira H. W. Dissanayake

**Affiliations:** ^1^Human Genetics Unit, Faculty of Medicine, University of Colombo, Sri Lanka; ^2^Faculty of Medicine and Allied Sciences, Rajarata University of Sri Lanka, Sri Lanka; ^3^Asiri Centre for Genomic and Regenerative Medicine, Asiri Surgical Hospital, Colombo, Sri Lanka

## Abstract

Translocations are the most common type of structural chromosomal abnormalities. Unbalanced translocations are usually found in children who present with congenital abnormalities, developmental delay, or intellectual disability. Balanced translocations are usually found in adults who frequently present with reproductive failure; either subfertility, or recurrent pregnancy loss. Herein, we report the spectrum and frequency of translocations in a Sri Lankan cohort. A database of patients undergoing cytogenetic testing was maintained prospectively from January 2007 to December 2016 and analyzed, retrospectively. A total of 15,864 individuals were tested. Among them, 277 (1.7%) had translocations. There were 142 (51.3%) unbalanced translocations and 135 (48.7%) balanced translocations. Majority (160; 57.8%) were Robertsonian translocations. There were 145 (52.3%) children and adolescents aged less than 18 years with translocations, and 142 (97.9%) were unbalanced translocations. Majority [138 (95.2%)] were referred due to congenital abnormalities, developmental delay, or intellectual disability, and 91 were children with translocation Down syndrome. All adults aged 18 years or above (132) had balanced translocations. Subfertility and recurrent pregnancy loss [84 (63.6%)] and offspring(s) with congenital abnormalities [48 (36.4%)] were the most common indications in this group. Majority (68.2%) in this group were females with reciprocal translocations (55.3%). Chromosomes 21, 14, and 13 were the most commonly involved with rob(14q21q) [72 (26%)], rob(21q21q) [30 (13.7%)], and rob(13q14q) [34 (12.3%)] accounting for 52% of the translocations. Chromosomes 1, 8, 11, and 18 were most commonly involved in reciprocal translocations. The observed high frequency of chromosomal translocations in our cohort highlights the importance of undertaking cytogenetic evaluation and providing appropriate genetic counseling for individuals with the phenotypes associated with these translocations.

## 1. Introduction

Chromosomal translocations refer to exchange of chromosomal segments between chromosomes. Translocations are the most common type of structural chromosomal abnormalities seen in the general population, having a frequency of about 1/1000 live births. Two types of chromosomal translocations are described: Robertsonian translocations and reciprocal translocations [1]. In Robertsonian translocations, the breakpoints commonly occur in the short arms of two acrocentric chromosomes (homologous or nonhomologous) with subsequent fusion and formation of dicentric chromosomes. It is reported that either the inactivation of one centromere or the close proximity of the two functional centromeres in these dicentric chromosomes allows them to remain stable [2]. Less frequent forms of Robertsonian translocations may be caused by breakage and fusion of centromeres (centric fusion) or from breakage and fusion of one short arm and one long arm of acrocentric chromosomes, resulting in monocentric rearrangements [3, 4]. In Robertsonian translocations, the loss of gene-poor short arms of the two acrocentric chromosomes usually does not produce any phenotypic effects. Most of these individuals remain undetected until they attempt to reproduce. Reciprocal translocations result from breakage of two nonhomologous chromosomes, with at least one of them being a nonacrocentric chromosome, and interchange of chromosomal fragments between them. Consequently, two derivative chromosomes are formed with no loss or gain of genetic material. Unless one or both of the chromosomal breakpoints involve an important functional gene, these balanced chromosomal rearrangements would not produce a significant phenotypic effect either. However, the carriers of both Robertsonian and reciprocal translocations commonly present with reproductive problems, due to unbalanced chromosomal segregation in meiosis, which cause significant chromosomal imbalances (i.e., disomies and nullisomies) in their gametes with subsequent partial aneuploidies in the conceptuses. This can lead to infertility, recurrent miscarriages, or offspring with congenital anomalies due to the unbalanced translocations.

Although all human chromosomes are theoretically susceptible to chromosomal breakage leading to translocations, the frequency of chromosomal translocations shows a nonrandom distribution. Translocations between acrocentric chromosomes 13 and 14 and rob(13q14q) constitute the majority of balanced Robertsonian translocations, with rob(14q21q) being the second most common type. Unbalanced Robertsonian translocations involving chromosome 21 are by far the commonest type of structural abnormalities giving rise to translocation Down syndrome. Other types of homologous or heterologous Robertsonian translocations are comparatively rare [5, 6]. A large number of case reports describing children or fetuses with congenital malformations, dysmorphic features, impaired growth, and/or development due to unbalanced reciprocal translocations involving different chromosomes have been reported in the scientific literature. The phenotypic features observed in these cases were attributed to partial monosomy and/or partial trisomy of different chromosomal segments that frequently occurred due to unbalanced chromosomal segregation during meiosis in a parent who is a carrier of a balanced reciprocal translocation.

It is also known that chromosomal translocations are one of the commonest types of inherited chromosomal abnormalities leading to recurrent pregnancy loss [7, 8]. Cytogenetic studies on male infertility have also showed a higher frequency of chromosomal translocations among infertile men than in the general population [9, 10]. It has been reported that the presence of a chromosomal translocation causes partial or complete spermatogenic arrest, with consequential oligospermia or azoospermia. In contrast, in female carriers of these chromosomal translocations, oogenesis is known to progress without an arrest in meiosis, resulting in the production of abnormal oocytes with unbalanced chromosomal constitution and subsequent partial aneuploidies in the conceptus [11].

In Sri Lanka, congenital malformations, dysmorphism, developmental delay, and intellectual disability are prevailing problems among children. Congenital abnormalities accounts for 43% of infant deaths and 47% of deaths in children aged 1-5 years, thus making it the leading cause of mortality in both groups [12]. However, the aetiological factors leading to these abnormalities in Sri Lankan children have not been studied extensively. Furthermore, the anecdotal evidence suggests that subfertility and recurrent pregnancy loss affect a large number of couples in Sri Lanka, yet country-wide frequency data are not available. Both congenital abnormalities in children and reproductive failure in adults are associated with Robertsonian and reciprocal translocations. Although, there are many studies conducted in Caucasian and other Asian populations describing the frequency of these chromosomal translocations and their associated phenotypes, there is paucity of data regarding the pattern of Robertsonian and reciprocal translocations and the associated phenotypes in the Sri Lankan population. This study aims to describe the frequency and pattern of Robertsonian and reciprocal translocations in a cohort of Sri Lankan patients referred for cytogenetic testing during a 10-year period. Such knowledge would be beneficial for providing accurate genetic counseling to reduce the burden of chromosomal disorders in children and subfertility and pregnancy losses in the country.

## 2. Materials and Methods

The sex, age, indication for referral, and the types of chromosomal translocations in patients referred for cytogenetic analysis to the Human Genetics Unit, Faculty of Medicine, University of Colombo and Asiri Centre for Genomic and Regenerative Medicine, Colombo, are maintained in anonymized databases completely delinked from the original patients. The data from January 2007 to December 2016 were retrospectively analyzed. Standard descriptive statistics were used to describe the characteristics of the study population and the frequency and distribution of the chromosomal translocations. The study protocol was approved by the Ethics Review Committee (ERC), Faculty of Medicine, University of Colombo, Sri Lanka [ERC protocol no. EC-16-142].

In all cases, chromosomal analysis had been performed on cultured lymphocytes from peripheral blood samples. Following trypsin-Giemsa banding, twenty metaphase spreads had been analyzed routinely in each sample. In case of suspected mosaicism, 40 metaphase spreads had been analyzed. Banding resolution of 550 was used routinely for the analysis. The reporting was done according to the guidelines of the International System for Human Cytogenetic Nomenclature (ISCN) 2016. All the adult patients and the parents or guardians of all the children tested had provided written consent for cytogenetic testing.

During the analysis the individuals with translocations were categorized in to 2 groups. "Children and adolescents" refer to individuals aged less than 18 years. Those who are aged 18 years or above at the time of referral were referred to as "adults".

## 3. Results

**Figure 1:**
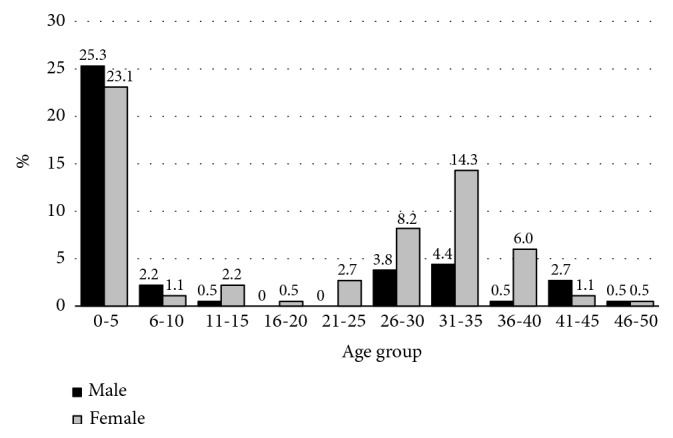
Distribution of the age of the individuals at the time of cytogenetic testing.

A total of 15,864 individuals were tested. Among them 277 (1.7%) had translocations. This accounts for 50.5% of the total number of structural chromosomal abnormalities (n=548), making translocations the most common type of structural chromosomal abnormalities in this group. There were 161 (58.1%) females and 116 (41.9%) males with translocations. Their ages ranged from 2 days old to 47 years, and 132 (47.7%) were adults. There were 145 (52.3%) children and adolescents, among them only 5 were above 12 years of age. The highest number of referrals for cytogenetic testing were recorded during the first 5 years of life (48.4%), followed by the age groups 31-35 years (18.7%) and 26-30 years (12.0%) (see Figure 1).

**Figure 2:**
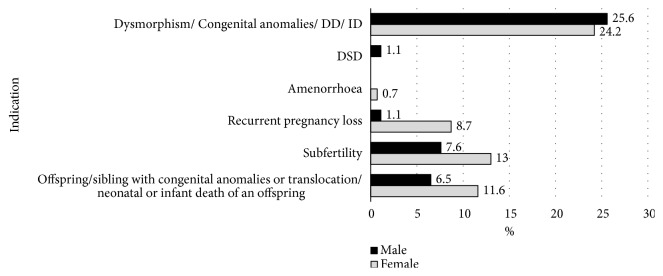
*Indications for the referral for cytogenetic testing among the individuals with a translocation.* DD: developmental delay, DSD: disorders of sex development, ID: intellectual disability, and RPL: recurrent pregnancy loss.

The commonest indications for cytogenetic testing were one or more of the following: dysmorphic features, congenital abnormalities, developmental delay, and intellectual disability (138, 49.8%). This group included children referred with the clinical diagnosis of aneuploidy syndromes, primarily Down syndrome. Subfertility was the commonest indication for testing (57, 20.6%) among the adults with a chromosomal translocation. Forty-eight adults (17.3%) were tested due to the family history (i.e., an offspring or a sibling) of a known translocation or congenital malformations or due to neonatal or infant death of an offspring, while 27 (9.7%) were tested due to recurrent pregnancy losses (see Figure 2).

Of the 277 translocations, 142 (51.3%) were unbalanced translocations which were detected exclusively among children and adolescents. All the adults (132) and 3 children (total 135; 48.7%) had balanced translocations. 160 (57.8%) were Robertsonian translocations, and 101 (63.1%) were detected among children and adolescents. Of the 117 reciprocal translocations, which account for 42.2% of all the translocations, 73 (62.3%) were detected in adults.

**Figure 3:**
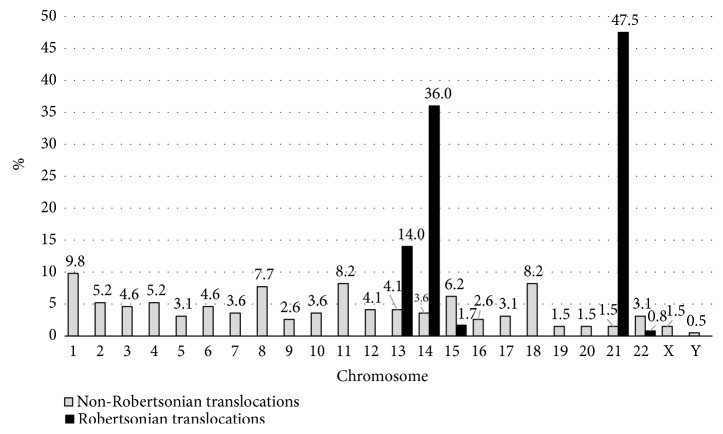
Distribution of the involvement of different chromosomes in Robertsonian and reciprocal translocations.

Involvement of chromosomes in reciprocal translocations showed a random distribution, with all the chromosomes involved in translocations at least on one occasion. Chromosomes 1, 18, 11, and 8 were most commonly involved with the frequencies of 9.0%, 9.0%, 7.7%, and 7.3%, respectively (Figure 3). The commonest reciprocal translocations were t(8;11), t(8;18), t(11;18), t(2;12), and t(7;14) in descending order of frequency. Chromosomes 19 (2.1%) and 20 (1.3%) were the least involved autosomes in translocations. There were only 4 autosome-sex chromosome translocations: t(X;1), t(X;21), t(X;22), and t(Y;15). Unlike the reciprocal translocations, involvement of acrocentric chromosomes in Robertsonian translocations showed a nonrandom distribution. Chromosome 21 was involved in 50% of these translocations followed by chromosome 14 (33.4%) and chromosome 13 (14.1%). Involvement of chromosomes 15 and 22 in Robertsonian translocations was uncommon (1.9% and 0.6%, respectively) (see Figure 3). Among the 160 Robertsonian translocations, rob(14q21q) was the most common type (72; 45%) followed by rob(21q21q) and rob(13q14q) (see Table 1). These 3 types of translocations account for 52% of all the translocations in this cohort of individuals.

### 3.1. Translocations in Children and Adolescents

Of the 145 children and adolescents with translocations, 74 (51.0%) were males and 71 (49.0%) were females. The age at referral ranged from 2 days old to 16 years, with a mean age of 2.0±3.6 years. There was an excess of Robertsonian translocations (101; 69.7%) compared to reciprocal translocations (44; 30.3%). Of the 101 children with Robertsonian translocations, 91 had translocation Down syndrome. Three other children had a complex karyotype with a Robertsonian translocation and a complete trisomy [46,XY,rob(13;14)(q10;q10),+21, 46,XX,rob(13;14)(q10;q10),+21 and 46,XX,+13,rob(13;14)(q10;q10)]. Four other children had an apparently balanced Robertsonian translocation [45,XX,rob(13;14)(q10;q10), 45,XY,rob(13;14)(q10;q10), 45,XY,rob(13;13)(q10;q10), and 45,XX,rob(15;21)(q10;q10)] but were referred for cytogenetic testing due to abnormal phenotype with one or more of the following: dysmorphic features, congenital malformations, developmental delay, and/or intellectual disability. Having a parent with a translocation was the reason for testing in 3 children with Robertsonian translocations, who were found to be the carriers of balanced translocations. Of the 44 children with unbalanced reciprocal translocations, the referral indications in 39 children were one or more of the following: dysmorphic features, congenital malformations, developmental delay, and intellectual disability. Three male children who were detected to have reciprocal translocations were referred due to disorders of sex development. Primary amenorrhoea was the referral indication of a 12-year-old girl and a 15-year-old girl who had translocations involving one of the X chromosomes [karyotypes 46,X,t(X;22)(p21.3;q10) and 46,X,t(X;1)(q13.1;p36.3)]. Another 2-year-old female child with an X-autosome translocation [karyotype 45,X,t(X;21)(q23;q22)] was referred for cytogenetic testing due to multiple congenital abnormalities (see Table 2).

 
46,XX,+13,rob(13;21)(q10;q10)

### 3.2. Translocations in Adults

Total of 132 adults were found to have chromosomal translocations. There were more females (90; 68.2%) than males (42; 31.8%) in this cohort. Their ages at the time of referral ranged between 20 years to 47 years with a mean age of 32.7±5.4 years. All of them were carriers of balanced translocations. There was an excess of reciprocal translocations in adults [73 (55.3%) versus 59 (44.7%)]. Commonest indication for cytogenetic testing in this group was subfertility [57 (20.6%)]. Twenty-seven (9.7%) adults with translocations were referred for testing due to recurrent pregnancy loss. Others [48 (36.4%)] were tested because of an offspring with a known chromosomal abnormality or congenital abnormalities or neonatal/infant death of an offspring. Among adults with either subfertility or recurrent pregnancy loss, rob(13q14q) was the commonest type of translocation, which was detected in 19 females and 7 males and accounted for 35.1% of translocations in the subfertility group and 22.2% of the translocations in the recurrent pregnancy loss group (see Tables 3 and 4). Robertsonian translocation rob(14q21q) which was detected more commonly in females (14 versus 2) was the commonest type of translocation (33.3%) in adults with an offspring with a translocation or congenital abnormality or neonatal or infant death of an offspring (see Table 5). Overall, rob(13q14q) was the commonest translocation (29; 22.0%) among the adults in this cohort followed by rob(14q21q), which was detected in 22 (16.7%) adults including 2 females with the 45,XX,rob(14q21q)/46,XX mosaicism.

## 4. Discussion

This is the first report of the frequency and spectrum of chromosomal translocations in the Sri Lankan population. Furthermore, to the best of our knowledge, we report the largest collection of chromosomal translocations in a South Asian population. Herein, we ratified the fact that translocations are the commonest type of structural chromosomal abnormalities. The frequency of translocations in our cohort (1.7%) was higher than the estimated worldwide population prevalence of 1/1000. This is probably because this study included individuals who were referred for cytogenetic testing with a clinical suspicion of an underlying chromosomal abnormality. The higher incidence of translocations in this study could also be linked with the high prevalence of congenital malformations reported among infants and children in Sri Lanka. Termination of pregnancies due to fetal indications is legally prohibited in Sri Lanka. This inevitably contributes to the higher prevalence of children born with these chromosomal abnormalities. Cytogenetic surveys of different populations with a study design similar to the current study which included selected populations with suspected chromosomal abnormalities have reported a comparable incidence of chromosomal translocations around 1-2%. These studies have also shown that translocations are the commonest structural chromosomal abnormalities in their populations [13–16].

Our observation of the nonrandom distribution of Robertsonian translocations is consistent with the existing published literature. A recent large-scale study of Robertsonian translocations in China reported rob(13q14q) as the commonest Robertsonian translocation, followed by rob(14q21q) [6]. In the present study, rob(14q21q) and rob(21q21q) commonly observed among children with Down syndrome were more frequent than rob(13q14q). It was suggested that the presence of homologous pericentric regions in chromosomes 13, 14, and 21 contributes to the higher incidence of translocations between these chromosomes [5]. Among the balanced reciprocal translocations, translocations involving chromosomes 11 and 22 are commonly described in the scientific literature [17, 18]. In a study of 269 balanced translocations among patients with recurrent miscarriages, there was a surplus of chromosomes 6, 7, and 22 in reciprocal translocations [19]. Another study with similar design showed an excess of chromosome 7 and 4 in reciprocal translocations [20]. In this study also chromosome 11 was one of the most commonly involved chromosomes in translocations; however, involvement of chromosomes 4, 6, and 7 was uncommon and chromosome 22 was one of the least commonly involved chromosomes. Herein we report a larger collection of reciprocal translocations than that reported in the scientific literature. A random involvement of chromosomes in reciprocal translocations was observed.

### 4.1. Translocations in Children and Adolescents

Large-scale cytogenetic surveys of newborn infants in different populations have reported translocations as one of the commonest autosomal abnormalities, second only to complete trisomy 21. The reported incidence of translocations ranges between 1 per 500 and 1 per 750 children (0.14%-0.18%) [21–23]. These studies reported a comparable incidence of Robertsonian and reciprocal translocations; however the number of translocations in each study was low [21, 22]. We report a much larger collection of translocations in children and adolescents, and Robertsonian translocations were found to account for more than two-thirds of the total number of translocations. This is probably due to the presence of a large number of children referred with the clinical diagnosis of Down syndrome who were found to have translocation Down syndrome, in comparison to the nonselective samples studied in the cytogenetic surveys mentioned above. Almost all the children with unbalanced Robertsonian translocations were diagnosed with Down syndrome except one neonate with Patau syndrome having the karyotype 46,XX,+13,rob(13;14)(q10;q10); a finding similar to a large-scale study conducted in China [6]. A cytogenetic analysis of nearly 30,000 children with Down syndrome in England and Wales reported that 2.7% of the cases were due to Robertsonian translocations including rob(21q21q) [24]. A study conducted in our laboratories in 2015 showed a higher incidence (4.7%, 31/665) of Down syndrome due to Robertsonian translocations [25]. Herein, we expand the collection of cases to 91, of which majority were due to rob(14q21q) followed by rob(21q21q). These findings differ from a study that included 258 children with translocation Down syndrome, in which there was an excess of rob(21q21q) than rob(14q21q) (123 versus 108) [6]. The common understanding is that the balanced translocations carriers are phenotypically normal except for the problems with fertility and reproduction. However, there are reports of abnormal phenotypes in balanced translocations carriers, and a postulated reason is the uniparental disomy in them [26]. We also report 4 phenotypically abnormal children with apparently balanced Robertsonian translocations; however we do not have adequate clinical information or data from advanced genetic tests to determine whether these translocations were incidental findings or were related to the phenotypes observed. Majority of the children with reciprocal translocations had one or more of the following indications: dysmorphic features, congenital malformations, developmental delay, and intellectual disability. We did not describe the phenotypic features of these children in detail, because it was beyond the scope of this study. Translocation between the X chromosome and an autosome is a recognized cause of primary amenorrhoea [27–29], which is similar to the 2 patients with X-autosome translocations in our study. We also report 3 children with reciprocal translocations involving autosomes who were referred primarily due to disorders of sex development. It is likely that the breakpoints in the chromosomal translocations disrupt one of the many genes involved in sex determination and differentiation [30]. We do not have data from advanced genetic studies for these 3 children to determine the molecular genetic basis of the abnormality observed in them. These findings provide more evidence to emphasize the importance of undertaking cytogenetic evaluation in children with these clinical conditions.

### 4.2. Translocations in Adults

Carriers of balanced chromosomal translocations usually do not have recognizable phenotypic abnormalities in their childhood and commonly present for cytogenetic evaluation as adults with reproductive failure, either subfertility or recurrent pregnancy loss, or with offspring(s) with congenital abnormalities. Consistent with this fact, we observed that all the adults in our cohort had balanced translocations and could be grouped in to one of the above clinical categories. Studies in the scientific literature have reported chromosomal translocations, mainly the reciprocal translocations as the commonest type of structural chromosomal abnormalities in couples with recurrent spontaneous miscarriages [19, 20, 31, 32]. Similarly we also observed an excess of reciprocal translocations in our adult cohort. However, rob(13q14q) is the single commonest type of translocation in this group and is similar to the findings observed in a recent large-scale study on Robertsonian translocations in China [6]. The only study published in the scientific literature regarding the cytogenetic profile of subfertility and recurrent pregnancy loss in Sri Lankans reported only 2 cases of chromosomal translocations among 442 patients tested [33]. The present study greatly expands the existing knowledge regarding these common problems in the Sri Lankan population. In this group of adults with subfertility or recurrent pregnancy loss, over two-thirds of the individuals were females (Figure 2). A possible explanation for this observation is that males with chromosomal translocations commonly present with subfertility due to oligospermia or azoospermia as a result of spermatogenic arrest [11], and in our setting males with these problems are not routinely karyotyped unless there is a clinical suspicion of Kinefelter syndrome. In contrast, in females who carry these translocations, oogenesis is continued with the risk of producing oocytes with unbalanced chromosomal complements and subsequent recurrent spontaneous miscarriage of the conceptus [11]. This is likely to be the reason for the higher number of females with a chromosomal translocation with recurrent pregnancy loss and also with offspring(s) with a chromosomal abnormality, congenital malformation, or death in the neonatal period or infancy. Failure of assisted reproductive procedures such as* in vitro* fertilization (IVF) is also a known adverse reproductive outcome, when one or both partners are carriers of the balanced chromosomal translocation [34, 35]. We also had a considerable number of subfertile couples who were awaiting IVF or were referred for testing following a failed IVF.

## 5. Limitations

In this study, we have used the G-banding resolution of 550 as routine practice. In cases with unbalanced translocations which were observed among children, the exact chromosomal breakpoints and size of the unbalanced segments could not be accurately delineated due to the unavailability of higher resolution molecular cytogenetic studies such as chromosomal microarray at our centre. There were few apparently balanced chromosomal translocations detected by G-banding karyotype among few children with abnormal phenotypic features, but we could not perform microarray testing to determine whether these are truly balanced events. Another major limitation encountered during the retrospective analysis was the lack of detailed clinical information. This caused the grouping of several different indications such as dysmorphism, congenital malformations, developmental delay, and intellectual disabilities among children into a single group. Thus, a comprehensive genotype-phenotype correlation of some of the cases could not be done due to the lack of access to detailed clinical information. Parents with balanced translocations that had led to unbalanced translocations in the offspring were indicated as “mat” or “pat” or as “dn” in the case of de novo events. However, there were several children with translocation whose parental karyotypes were not available. Therefore, we could not determine whether these cases were inherited or de novo events and the estimation of the risk of recurrence of these translocations could not be ascertained.

## 6. Conclusions

We have documented, for the first time, the spectrum and frequency of chromosomal translocations in a Sri Lankan cohort and to the best of our knowledge this is the largest collection of chromosomal translocations in a South Asian population. As the main diagnostic centres providing these services in the country, patients referred from all parts of the country and from all ethnic groups in Sri Lanka are represented in this cohort. Hence, the results of this study can be applied to the whole population of the country to determine the burden of these chromosomal abnormalities and to plan out appropriate diagnostic, therapeutic, and preventive measures. The high frequency of chromosomal translocations in our cohort highlights the importance of undertaking cytogenetic evaluation in children with dysmorphic features, congenital malformations, developmental delay, and intellectual disability including those with clinically recognizable aneuploidy syndromes, abnormal sex development, delayed puberty, and primary amenorrhoea and in adults with subfertility and recurrent pregnancy loss. The cytogenetic diagnosis enables comprehensive genetic counseling, risk prediction, and prevention of these chromosomal abnormalities, thus reducing the burden of these disorders in the population. Even though advanced genetic testing facilities are required for the precise delineation of molecular genetic abnormalities and accurate diagnosis of the affected cases, routine cytogenetic testing through karyotyping plays a pivotal role in the diagnosis and management of these common clinical conditions, especially in low-resource settings where access to advanced molecular genetic testing services is unavailable.

## Figures and Tables

**Table 1:** Distribution of the different types of translocations in this study.

Chr	1	2	3	4	5	6	7	8	9	10	11	12	13	14	15	16	17	18	19	20	21	22	X	Y	Total
1	0	0	0	2	1	2	1	0	0	1	3	0	3	0	3	0	1	2	1	0	0	0	1	0	*21*
2		0	0	0	1	0	0	3	1	0	0	4	3	0	1	0	0	0	0	0	0	0	0	0	*13*
3			0	0	0	2	0	0	0	0	1	0	0	0	1	0	2	2	0	0	0	1	0	0	*9*
4				0	0	1	0	1	0	1	0	0	2	2	0	2	0	0	0	0	0	1	0	0	*10*
5					0	0	0	2	0	0	1	0	0	1	0	2	0	0	0	1	0	1	0	0	*8*
6						0	1	0	1	1	0	0	0	0	0	1	0	0	0	0	0	2	0	0	*6*
7							0	1	0	1	0	0	0	4	0	0	1	0	0	0	0	0	0	0	*7*
8								0	0	0	5	0	0	0	0	0	0	5	0	0	0	0	0	0	*10*
9									0	0	1	0	1	0	0	0	1	0	1	0	1	0	0	0	*5*
10										0	1	0	0	0	1	0	1	0	0	0	0	0	0	0	*3*
11											0	0	0	0	0	0	1	4	0	0	0	1	0	0	*6*
12												0	0	0	3	0	0	1	2	0	0	0	0	0	*6*
13													**1**	**34**	**1**	0	0	2	0	0	**7**	**1**	0	0	*46*
14														**0**	**0**	0	0	0	0	0	**72**	**1**	0	0	*73*
15															**0**	2	0	0	0	0	**5**	**0**	0	1	*8*
16																0	0	2	0	1	0	0	0	0	*3*
17																	0	0	0	0	0	0	0	0	*0*
18																		1	0	1	0	0	0	0	*2*
19																			0	0	1	0	0	0	*1*
20																				0	0	0	0	0	*0*
21																					**38**	**0**	1	0	*39*
22																						**0**	1	0	*1*
X																							0	0	*0*
Y																								0	*0*

*Total*	*0*	*0*	*0*	*2*	*2*	*5*	*2*	*7*	*2*	*4*	*12*	*4*	*10*	*41*	*10*	*7*	*7*	*19*	*4*	*3*	*124*	*8*	*3*	*1*	*277*

Chr: chromosome. Robertsonian translocations are indicated in the cells with bold font.

**Table 2:** Chromosomal translocations in children and adolescents.

Indication	Karyotype	Number	(%)
*Unbalanced translocations*			

Down syndrome	*46,XX,rob(14;21)(q10;q10),+21mat*	4	(2.8)
	*46,XX,rob(14;21)(q10;q10),+21*dn	1	(0.7)
	*46,XX,rob(14;21)(q10;q10),+21∗*	15	(10.3)
	*46,XY,rob(14;21)(q10;q10),+21mat*	3	(2.1)
	*46,XY,rob(14;21)(q10;q10),+21*dn	4	(2.8)
	*46,XY,rob(14;21)(q10;q10),+21∗*	20	(13.8)
	*46,XX,+21,rob(21;21)(q10;q10)*dn	1	(0.7)
	*46,XX,+21,rob(21;21)(q10;q10)∗*	18	(12.4)
	*46,XY,+21,rob(21;21)(q10;q10)pat*	1	(0.7)
	*46,XY,+21,rob(21;21)(q10;q10)*dn	1	(0.7)
	*46,XY,+21,rob(21;21)(q10;q10)∗*	13	(9.0)
	*46,XX,rob(15;21)(q10;q10),+21∗*	2	(1.4)
	*46,XY,rob(15;21)(q10;q10),+21∗*	2	(1.4)
	*46,XX,rob(13;21)(q10;q10),+21*dn	1	(0.7)
	*46,XY,rob(13;21)(q10;q10),+21∗*	2	(1.4)
	*mos46,XX,+21,rob(21;21)(q10;q10)[7]/46,XX[12]∗*	1	(0.7)
	*mos46,XX,+21,rob(21;21)(q10;q10)[6]/46,XX[5]*dn	1	(0.7)
	*mos47,XY,+21[15]/46,XY,rob(14;21)(q10;q10),+21[10]∗*	1	(0.7)
	*46,XX,rob(13;14)(q10;q10),+21pat*	1	(0.7)
	*46,XY,rob(13;14)(q10;q10),+21pat*	1	(0.7)
	47,XY,+21,t(1;18)(q41;q12.1)*∗*	1	(0.7)
	mos45,XX,t(19;21)[2]/47,XX,+21[2]/46,XX[46]*∗*	1	(0.7)

Edward syndrome	47,XX,+18,t(16;18)(q24;12.2)mat	1	(0.7)

Patau syndrome	*46,XX,+13,rob(13;14)(q10;q10)pat*	1	(0.7)
	46,XY,der(18)t(13;18)(q14;q23)mat	1	(0.7)

DSD	46,XY,t(6;22)(q16.3;q13.2)mat	1	(0.7)
	46,XY,t(6;9)(p11.1;p11)*∗*	1	(0.7)
	46,XY,t(9;11)(p24;q22)*∗*	1	(0.7)

Primary Amenorrhoea	46,X,t(X;1)(q13.1;p36.3)*∗*	1	(0.7)
	46,X,t(X;22)(p21.3;q10)*∗*	1	(0.7)

Dysmorphism/congenital malformations/developmental delay/intellectual disability			

	*45,XX,rob(13;14)(q10;q10)∗*	1	(0.7)
	*45,XY,rob(13;14)(q10;q10)∗*	1	(0.7)
	*45,XX,rob(15;21)(q10;q10)∗*	1	(0.7)
	*45,XY,rob(13;13)(q10;q10)∗*	1	(0.7)
	46,XY,t(2;8)(p22;p23.3)pat	2	(1.4)
	46,XX,der(16)t(5;16)(q33;q24)mat	2	(1.4)
	46,XY,der(18)t(8;18)(p21.3;p11.2)mat	2	(1.4)
	47,XY,+der(12)t(12;15)(p12;p13)mat	2	(1.4)

	Other reciprocal translocations	27	(18.6)

	46,XX,t(1;4)(q42;q35)mat	46,XX,t(7;14)(q11.1;q11.1)*∗*	
	46,XY,der(6)t(1;6)(p36.3;q23)pat	46,XX,der(14)t(7;14)(q22;q32)mat	
	47,XY,+der(15)t(1;15)(q44;q22)mat	46,XY,der(11)t(8;11)(p21;q25)pat	
	46,XY,t(1;19)*∗*	46,XY,t(8;11)(q24.3;p13)pat	
	46,XY,t(2;12)(q34;q13)*∗*	46,XX,t(8;18)(q24;q21)pat	
	46,XX,der(2)t(2;13)(q37.3;q13)mat	46,XX,t(9;21)(p24;p13)*∗*	
	46,XX,der(15)t(2;15)(q31;q11.1)*∗*	45,X,t(X;21)(q23;q22)dn	
	46,XY,der(3)t(3;18)(p26:q21.1)pat	46,XX,der(10)t(10;15)(q26;q21)*∗*	
	46,XY,t(4;6)(q22;p25)*∗*	46,XY,der(11)t(11;18)(q25;q12.1)pat	
	46,XY,der(4)t(4;13)(q21;q10)mat	46,XX,der(15)t(15;16)(p13;q21)mat	
	47,XY,+21,der(16)t(4;16)(q31.1;q24)pat	46,XX,der(18)?t(18;18)(q11.2;q11.2)*∗*	
	46,XX,der(5),t(5;8),(p13;q13)pat	46,XX,t(18;20)(q21.1;p13)*∗*	
	46,XX,t(5;11)(q31;q23)*∗*	mos46,XX,der(22)t(5;22)(p12;q11.2)[6]/46,XX[10]*∗*	
	46,XY,t(5;14)(q13.2;q32.3)mat		
*Balanced translocations*			

Sibling/parent with a chromosomal translocation			

	*45,XX,rob(14;21)(q10;q10)mat*	1	(0.7)
	*45,XX,rob(14;21)(q10;q10)∗*	1	(0.7)
	*45,XX,rob(13;21)(q10;q10)∗*	1	(0.7)

*Total*	*145*		

DSD: disorder of sex development.

Robertsonian translocations are in italic text.

dn: *de novo.*

pat: paternal.

mat: maternal.

*∗*Parental karyotypes were not available.

**Table 3:** Chromosomal translocations in patients referred for subfertility.

Karyotype		Number	(%)
*Robertsonian translocations*			
45,XX,rob(13;14)(q10;q10)		13	(22.8)
45,XY,rob(13;14)(q10;q10)		7	(12.3)
45,XX,rob(14;21)(q10;q10)		2	(3.5)
45,XY,rob(14;21)(q10;q10)		1	(1.8)
mos45,XX,rob(14;21)(q10;q10)[20]/46,XX[12]		1	(1.8)
		*24*	

*Reciprocal translocations*			
46,XX,t(1;4)(q43;q44)	46,XX,t(4;10)(q32;q23)	*33*	(57.9)
46,XY,t(1;7)(q24;q36)	46,XX,t(4;14)(q12;q32)		
46,XX,t(1;11)(p35;q13.3)	46,XY,t(5;20)(q23.3;p13)		
46,XX,t(1;11)(q41;q22)	46,XY,t(6;7)(p23;p15.3)		
46,XX.t(1;11)(q41;q14)	46,XX,t(6;10)(p12;p12.1)		
46,XX,t(1;13)(q22;q32)	46,XX,t(7;8)(p22;q22.3)		
46,XY,t(2;5)(q34;q35.3)	46,XX,t(7;17)(p13;p13)		
46,XX,t(2;12)(p10;p10)	46,XX,t(8;11)(p12;q25)		
46,XX,t(2;12)(p21;p12)	46,XY,t(9;19)(p22;q13.2)		
46,XY,t(2;12)(q21;q24.1)	46,XX.t(10;11)(q22;p15)		
46,XY,t(2;13)(q21;q34)	46,XX,t(11;17)(q13.1;q13.3)		
46,XX,t(3;6)(p21.2;q27)	46,XY,t(11;18)(q13.1;q23)		
46,XX,t(3;11)(p21.2;q25)	46,XX,t(12;18)(q21.3;q23)		
46,XY,t(3;15)(q10;q10)	46,XX,t(12;19)(q24.2;q13.4)		
46,XY,t(3;17)(q21.3;q25)	46,XY,t(12;19)(q22;q13.4)		
46,XX,t(3;22)(p22;q12)	46,XY,t(Y;15)(q12;p11.3)		
46,XY,t(4;8)(q31;q24)			

*Total*		*57*	

**Table 4:** Chromosomal translocations in patients referred for recurrent pregnancy loss.

Karyotype		Number	(%)
*Robertsonian translocations*			
45,XX,rob(13;14)(q10;q10)		6	(22.2)
45,XX,rob(14;21)(q10;q10)		1	(3.7)
45,XX,rob(13;15)(q10;q10)		1	(3.7)
45,XX,rob(13;21)(q10;q10)		2	(7.4)
45,XX,rob(13;22)(q10;q10)		1	(3.7)
45,XX,rob(14;22)(q10;q10)		1	(3.7)
		*12*	

*Reciprocal translocations*			
46,XX,t(1;5)(p36.1;p15.2)	46,XX,t(6;16)(p24;q12)	*15*	(55.5)
46,XY,t(1;13)(q44;q14.1)	46,XX,t(7;10)(q22;q23.3)		
46.XX,t(1;13)(q31;q14)	46,XX,t(7;14)(q36;q11.2)		
46,XY,t(1;15)(p36.3;q26.1)	46,XY,t(9;17)(q32;q13)		
46,XX,t(2;9)(p25.3;q34.1)	46,XX,t(10;17)(q23.3;q13)		
46,XX,t(3;6)(p22;q23)	46,XX,t(12;15)(p12;p13)		
46,XX,t(3;17)(p25;q25)	46,XX,t(16;20)(p13.3;q13.2)		
46,XX,t(3;18)(q24;q12.3)			

*Total*		*27*	

**Table 5:** Chromosomal translocations in patients referred due to an offspring with a translocation or congenital abnormalities or neonatal/infant death of an offspring.

Karyotype		Number	(%)
*Robertsonian translocations*			
45,XX,rob(14;21)(q10;q10)		14	(29.2)
45,XY,rob(14;21)(q10;q10)		2	(4.2)
mos45,XX,rob(14;21)(q10;q10)[10]/46,XX[15]		1	(2.1)
45,XY,rob(13;14)(q10;q10)		3	(6.3)
45,XX,rob(21;21)(q10;q10)		1	(2.1)
45,XY,rob(21;21)(q10;q10)		1	(2.1)
45,XX,rob(13;21)(q10;q10)		1	(2.1)
		*23*	(47.9)

*Reciprocal translocations*			
46,XY,t(1;6)(p36.3;q23)	46,XX,t(7;14)(q22;q32)	*25*	(52.1)
46,XY,t(1;10)(q12;p11.2)	46,XY,t(8;11)(p21;q25)		
46,XX,t(1;15)(q44;q22)	46,XY,t(8;11)(q24.3;p13)		
46,XX,t(1;17)(p36.3;q22)	46,XX,t(8;18)(p21.3;p11.23)		
46,XY,t(1;18)(q32;q11.2)	46,XY,t(8;18)(q26.1;q21.1)		
46,XY,t(2;8)(p22;p23.3)	46,XX,t(9;13)(p22;q33)		
46,XX,t(2;13)(q37.3;q13)	46,XY,t(11;18)(q25;q12.1)		
46,XX,t(4;13)(q21;q10)	46,XY,t(11;18)(q25;q12.1)		
46,XY,t(4;14)(p16;q32)	46,XX,t(11;22)(q23.3;q11.2)		
46,XY,t(4;16)(q31.1;q24)	46,XX,t(13;18)(q14;q23)		
46,XX,t(4;22)(p11.1;p13)	46,XX,t(15;16)(p13;q21)		
46,XY,t(5;8)(p13;q13)	47,XX,t(16;18)(q24;12.2)		
46,XX,t(6,22)(q16.3;q13.2)			

*Total*		*48*	

## Data Availability

The data used to support the findings of this study are included within the article.

## References

[1] Lubs H. A., Ruddle F. H. (1970). Chromosomal abnormalities in the human population: estimation of rates based on new haven newborn study. *Science*.

[2] Kim S.-R., Shaffer L. G. (2002). Robertsonian translocations: mechanisms of formation, aneuploidy, and uniparental disomy and diagnostic considerations. *Genetic Testing*.

[3] Daniel A., Lam Po Tang P. R. L. C. (1976). Structure and inheritance of some heterozygous robertsonian translocations in man. *Journal of Medical Genetics*.

[4] Page S. L., Shin J.-C., Han J.-Y., Choo K. H. A., Shaffer L. G. (1996). Breakpoint diversity illustrates distinct mechanisms for robertsonian translocation formation. *Human Molecular Genetics*.

[5] Therman E., Susman B., Denniston C. (1989). The nonrandom participation of human acrocentric chromosomes in Robertsonian translocations. *Annals of Human Genetics*.

[6] Zhao W.-W., Wu M., Chen F. (2015). Robertsonian translocations: an overview of 872 robertsonian translocations identified in a diagnostic laboratory in China. *PLoS ONE*.

[7] Ford HB., Schust DJ. (2009). Recurrent pregnancy loss: etiology, diagnosis, and therapy. *Reviews in Obstetrics and Gynecology*.

[8] Chaithra P. T., Malini S. S., Sharath Kumar C. (2011). An overview of genetic and molecular factors responsible for recurrent pregnancy loss. *International Journal of Human Genetics*.

[9] Egozcue S., Blanco J., Vendrell J. M. (2000). Human male infertility: chromosome anomalies, meiotic disorders, abnormal spermatozoa and recurrent abortion. *Human Reproduction Update*.

[10] Dong Y., Du R.-C., Jiang Y.-T., Wu J., Li L.-L., Liu R.-Z. (2012). Impact of chromosomal translocations on male infertility, semen quality, testicular volume and reproductive hormone levels. *Journal of International Medical Research*.

[11] Hunt P. A., Hassold T. J. (2002). Sex matters in meiosis. *Science*.

[12] (2013). Family Health Bureau. *Ministry of Health, Sri Lanka*.

[13] Kim H. J., Jung S. C., Moon H. R. (1999). Chromosome abnormalities in a referred population for suspected chromosomal aberrations. *Journal of Korean Medical Science*.

[14] Duarte A. C., Cunha E., Roth J. M., Ferreira F. L. S., Garcias G. L., Martino-Roth M. G. (2004). Cytogenetics of genetic counseling patients in Pelotas, Rio Grande do Sul, Brazil. *Genetics and Molecular Research*.

[15] Balkan M., Akbas H., Isi H. (2010). Cytogenetic analysis of 4216 patients referred for suspected chromosomal abnormalities in Southeast Turkey. *Genetics and Molecular Research*.

[16] Rajasekhar M., Murugesan R., Rekharao (2010). Cytogenetic analysis of 1400 referral cases: Manipal experience. *International Journal of Human Genetics*.

[17] Fraccaro M., Lindsten J., Ford C. E. (1980). The 11q;22q translocation: A European collaborative analysis of 43 cases. *Human Genetics*.

[18] Iselius L., Lindsten J., Aurias A. (1983). The 11q;22q translocation: a collaborative study of 20 new cases and analysis of 110 families. *Human Genetics*.

[19] Campana M., Serra A., Neri G. (1986). Role of chromosome aberrations in recurrent abortion: A study of 269 balanced translocations. *American Journal of Medical Genetics*.

[20] Kochhar P. K., Ghosh P. (2013). Reproductive outcome of couples with recurrent miscarriage and balanced chromosomal abnormalities. *Journal of Obstetrics and Gynaecology Research*.

[21] Jacobs P. A., Melville M., Ratcliffe S., Keay A. J., Syme J. (1974). A cytogenetic survey of 11,680 newborn infants. *Annals of Human Genetics*.

[22] Hamerton J. L., Canning N., Ray M., Smith S. (1975). A cytogenetic survey of 14,069 newborn infants. *Clinical Genetics*.

[23] Nielsen J., Wohlert M. (1991). Chromosome abnormalities found among 34910 newborn children: results from a 13-year incidence study in Arhus, Denmark. *Human Genetics*.

[24] Morris J. K., Alberman E., Mutton D., Jacobs P. (2012). Cytogenetic and epidemiological findings in Down syndrome: England and Wales 1989–2009. *American Journal of Medical Genetics Part A*.

[25] Thillainathan S., Sirisena N. D., Kariyawasam K. W. J. C., Jayasekara R. W., Dissanayake V. H. W. (2015). Cytogenetic analysis of chromosomal abnormalities in Sri Lankan children. *World Journal of Pediatrics*.

[26] Berend S. A., Bejjani B. A., McCaskill C., Shaffer L. G. (2002). Identification of uniparental disomy in phenotypically abnormal carriers of isochromosomes or Robertsonian translocations. *American Journal of Medical Genetics*.

[27] Vijayalakshmi J., Koshy T., Kaur H. (2010). Cytogenetic analysis of patients with primary amenorrhea. *International Journal of Human Genetics*.

[28] Ayatollahi H., Safaei A., Vasei M. (2010). Cytogenetic analysis of patients with primary amenorrhea in southwest of Iran. *Iranian Journal of Pathology*.

[29] Jiao X., Qin C., Li J. (2012). Cytogenetic analysis of 531 Chinese women with premature ovarian failure. *Human Reproduction*.

[30] Refai O., Friedman A., Terry L. (2010). De Novo 12;17 translocation upstream of sox9 resulting in 46,xx testicular disorder of sex development. *American Journal of Medical Genetics Part A*.

[31] Elghezal H., Hidar S., Mougou S., Khairi H., Saâd A. (2007). Prevalence of chromosomal abnormalities in couples with recurrent miscarriage. *Fertility and Sterility*.

[32] Ozawa N., Maruyama T., Nagashima T. (2008). Pregnancy outcomes of reciprocal translocation carriers who have a history of repeated pregnancy loss. *Fertility and Sterility*.

[33] Dissanayake V. H., Athapaththu A. M., Opanayake D. J., Hidellage N. T., Wijetunge K., Pedurupillay C. R. (2010). Chromosomal abnormalities in patients with recurrent spontaneous pregnancy loss and sub-fertility. *Sri Lanka Journal of Obstetrics and Gynaecology*.

[34] Stern C., Pertile M., Norris H., Hale L., Baker H. W. G. (1999). Chromosome translocations in couples with in-vitro fertilization implantation failure. *Human Reproduction*.

[35] Raziel A., Friedler S., Schachter M., Kasterstein E., Strassburger D., Ron-El R. (2002). Increased frequency of female partner chromosomal abnormalities in patients with high-order implantation failure after in vitro fertilization. *Fertility and Sterility*.

[36] Paththinige C. S., Sirisena U. N. D., Kariyawasam U. G. I. U., Dissanayake V. H. W. The frequency and phenotypic spectrum of cytogenetic abnormalities.

